# REM Sleep at its Core – Circuits, Neurotransmitters, and Pathophysiology

**DOI:** 10.3389/fneur.2015.00123

**Published:** 2015-05-29

**Authors:** Jimmy J. Fraigne, Zoltan A. Torontali, Matthew B. Snow, John H. Peever

**Affiliations:** ^1^Department of Cell and Systems Biology, University of Toronto, Toronto, ON, Canada

**Keywords:** REM sleep, brainstem, narcolepsy, cataplexy, hypothalamus, amygdala, dopamine, REM sleep behavior disorder

## Abstract

Rapid eye movement (REM) sleep is generated and maintained by the interaction of a variety of neurotransmitter systems in the brainstem, forebrain, and hypothalamus. Within these circuits lies a core region that is active during REM sleep, known as the subcoeruleus nucleus (SubC) or sublaterodorsal nucleus. It is hypothesized that glutamatergic SubC neurons regulate REM sleep and its defining features such as muscle paralysis and cortical activation. REM sleep paralysis is initiated when glutamatergic SubC cells activate neurons in the ventral medial medulla, which causes release of GABA and glycine onto skeletal motoneurons. REM sleep timing is controlled by activity of GABAergic neurons in the ventrolateral periaqueductal gray and dorsal paragigantocellular reticular nucleus as well as melanin-concentrating hormone neurons in the hypothalamus and cholinergic cells in the laterodorsal and pedunculo-pontine tegmentum in the brainstem. Determining how these circuits interact with the SubC is important because breakdown in their communication is hypothesized to underlie narcolepsy/cataplexy and REM sleep behavior disorder (RBD). This review synthesizes our current understanding of mechanisms generating healthy REM sleep and how dysfunction of these circuits contributes to common REM sleep disorders such as cataplexy/narcolepsy and RBD.

## Introduction

Rapid eye movement (REM) sleep is characterized by rapid eye movements, cortical activation, vivid dreaming, skeletal muscle paralysis (atonia), and muscle twitches (1–3). A distributed network of micro-circuits within the brainstem, forebrain, and hypothalamus is required for generating and sculpting REM sleep. This review will describe our current understanding of the cells and circuits that mediate REM sleep in both health and disease.

Disturbances in the normal control of REM sleep underlie cataplexy/narcolepsy and RBD, which are two common and serious sleep disorders. Narcoleptics not only experience pronounced sleep disturbances, but they also experience cataplexy – the sudden unwanted loss of muscle tone during otherwise normal wakefulness. Cataplexy is hypothesized to result from intrusion of REM sleep paralysis into wakefulness (4). By contrast, those with RBD suffer from the loss of normal muscle paralysis during REM sleep, which results in pathological levels of movement during REM sleep episodes. REM movements are often violent and forceful, and can result in bodily injury. Understanding the neural circuits that generate REM sleep and REM sleep paralysis is needed in order to clarify the pathophysiology of narcolepsy/cataplexy and RBD (4, 5). In this review, we discuss how REM sleep-control mechanisms underlie the intrusion of REM sleep paralysis during wakefulness in narcolepsy with cataplexy, and how degeneration of this same circuitry could underlie RBD. Finally, we discuss how newly identified hypothalamic circuits control REM sleep and how they potentially contribute to the pathophysiology of narcolepsy with cataplexy.

## The REM Sleep Core is Located in the Brainstem

**Figure 1:**
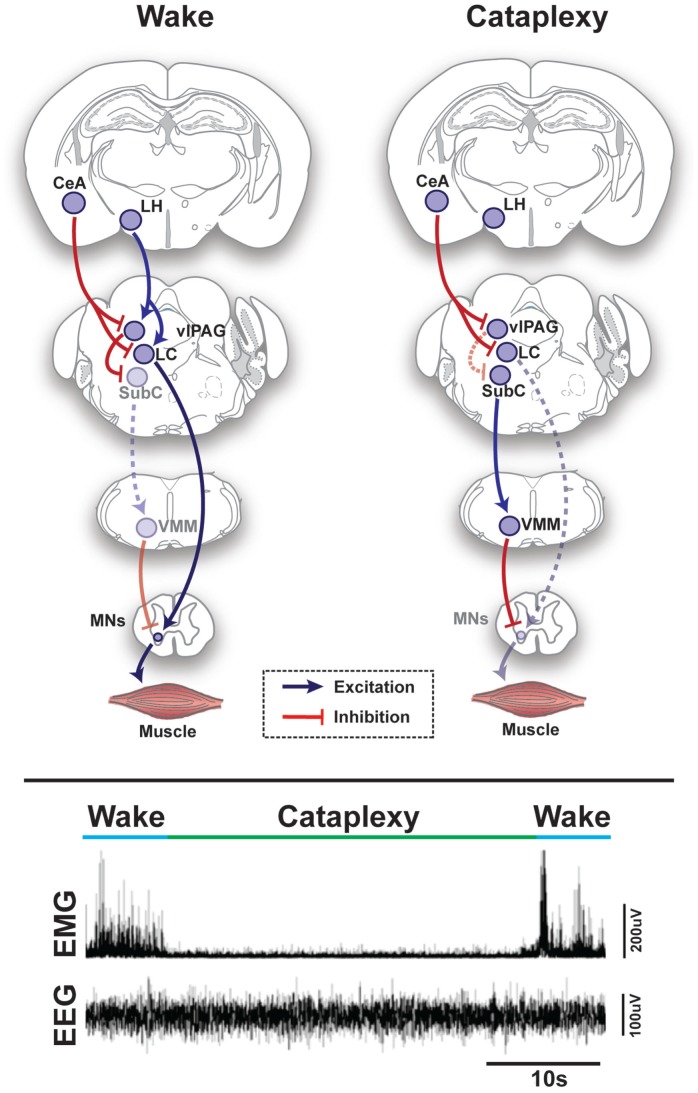
**Schematic representation of circuits and pathways regulating muscle activity during “normal” wakefulness and cataplexy in the rodent brain**. Inappropriate activation of rapid eye movement (REM) sleep muscle paralysis circuitry during wakefulness is thought to produce cataplexy. Glutamatergic REM-active SubC neurons trigger the paralysis of REM sleep via stimulation of the GABAergic/glycinergic cells in the VMM. These VMM neurons send inhibitory projections to skeletal motor neurons. Under normal condition, strong positive emotions are processed via GABAergic neurons of the CeA, which then inhibit cells in the LC and vlPAG. However, in the absence of the LH hypocretinergic neurons in cataplexy, this inhibition is left unbalanced and the REM sleep core circuit (i.e., SubC) is released from inhibition and triggers untimely muscle paralysis while the individual remains conscious. The inhibition of LC neurons during cataplexy removes noradrenergic inputs to motorneurons, thereby enhancing the muscle paralysis of cataplexy. Lower inset represents the brain (EEG) and muscle (EMG) activity in a narcoleptic mouse (i.e., orexin knockout mouse) at the transition into cataplexy [adapted from Burgess and Peever (17)]. Abbreviations: CeA, central nucleus of the amygdala; GABA, γ-aminobutyric acid; LC, locus coeruleus; LH, lateral hypothalamus; VMM, ventral medial medulla; SubC, subcoeruleus; vlPAG, ventrolateral periaqueductal gray; MNs, motoneurons; EEG, electroencephalogram; EMG, electromyogram; a.u., arbitrary unit.

**Figure 2:**
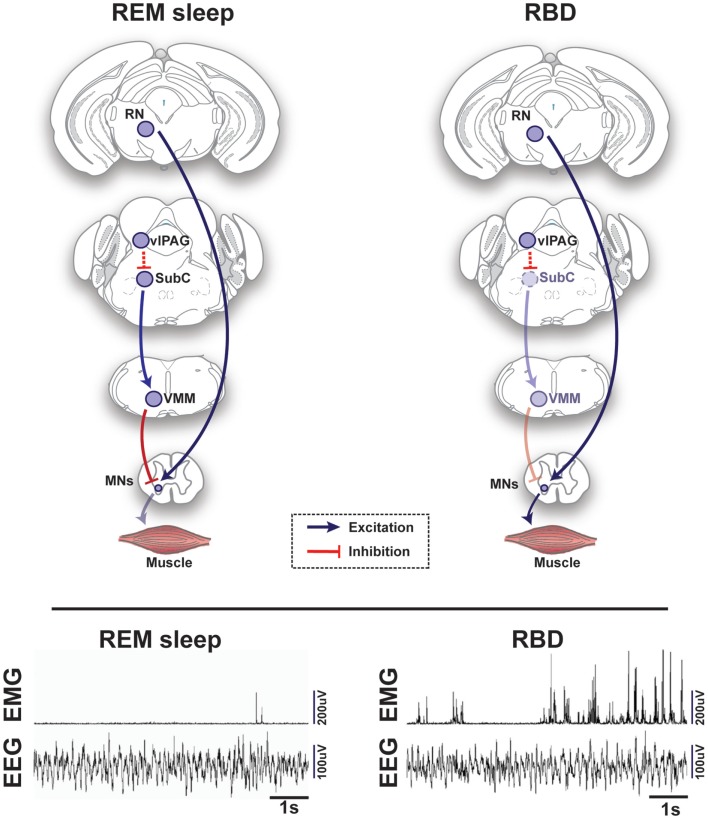
**Schematic representation of circuits and pathways regulating muscle activity during “normal” rapid eye movement (REM) sleep and REM sleep behavior disorder (RBD) in the rodent brain**. During REM sleep, REM-active glutamatergic SubC neurons trigger REM sleep paralysis through activation of GABAergic/glycinergic cells in the VMM, which carry inhibitory projections to skeletal motor neurons. Under normal REM sleep conditions, the SubC → VMM circuit inhibits motoneurons, which produces paralysis and limits the intrusion of muscle twitches and movement generated by the red nucleus (RN). However, in patients with RBD, degeneration of the SubC → VMM circuit releases motoneurons from their normal source of inhibition, which allows excitatory inputs to produce motor behaviors during REM sleep. Lower inset represents the brain (EEG) and muscle (EMG) activity during REM sleep in a healthy mouse (left) vs. a transgenic mouse model of RBD (right) [adapted from Brooks and Peever (18)]. Abbreviations: GABA, γ-aminobutyric acid; VMM, ventral medial medulla; SubC, subcoeruleus; vlPAG, ventrolateral periaqueductal gray; MNs, motoneurons; RN, red nucleus; EEG, electroencephalogram; EMG, electromyogram.

The core of the REM-generating circuit is localized at the mesopontine junction, medial to the trigeminal motor nucleus and ventral to the locus coeruleus (LC) (Figures 1 and 2) (6–8). The subcoeruleus nucleus (SubC), which is also called the sublaterodorsal nucleus, is composed of REM-active neurons – cells that are predominantly active during episodes of REM sleep (7–11). The majority of REM-active SubC cells are glutamatergic (12), suggesting that REM sleep is generated by a glutamatergic mechanism. However, GABA SubC cells have also been implicated in REM sleep control (8). Pharmacological activation of SubC cells can induce REM sleep motor atonia (6–13); whereas, SubC lesions can prevent REM sleep atonia and/or reduce REM sleep amounts (7, 8). SubC cells are thought to induce REM sleep muscle paralysis by recruiting GABA/glycine neurons in the ventromedial medulla (VMM) and spinal cord (Figures 1 and 2). These cells produce motor atonia during REM sleep by inhibiting skeletal motoneurons (8, 13–16).

Both GABA and glycine inhibition of motoneurons are required for producing REM sleep muscle paralysis (19–21). Pioneering intracellular recordings during REM sleep have shown that skeletal motoneurons are tonically hyperpolarized by large intracellular post-synaptic potentials. Local iontophoretic application of a glycine receptor antagonist (strychnine) diminishes this hyperpolarization, indicating that motoneurons are inhibited by a glycinergic mechanism during REM sleep (22, 23). Simultaneous antagonism of GABA_A_/GABA_B_/glycine receptors on motoneurons prevents REM sleep atonia, indicating that both GABA and glycine-mediated inhibition of motoneurons underlies REM sleep atonia (20, 21). However, acetylcholine also appears to suppress respiratory motoneuron activity during natural REM sleep (24–26). Loss or decrease in glutamatergic, noradrenergic, serotonergic, dopaminergic, and hypocretinergic activity during REM sleep may also function to reduce muscle activity and thereby contribute to the atonia of REM sleep (27–30).

Cholinergic REM-active neurons have been postulated to play a role in REM sleep initiation and control over motor atonia (31). Recently, it has been shown that acetylcholine activates spinally projecting SubC neurons (32). These cholinergic inputs into the SubC neurons mediate muscle atonia by both enhancing glutamate-driven post-synaptic excitation and facilitating pre-synaptic glutamate release. These results demonstrate that acetylcholine not only acts to directly entrain the core of the REM sleep circuitry, but also modulates the glutamatergic mechanisms that underlie REM sleep muscle control (7). Importantly, Grace and colleagues demonstrate that acetylcholine is not necessary to initiate the entrance into REM sleep; however, the cholinergic inputs to SubC neurons rather strengthen transitions once initiated (33). In this study, inactivation of cholinergic receptors increased the latency of NREM-to-REM sleep transitions and caused a greater proportion of these transitions to fail into entering REM sleep. Reciprocally, the activation of the cholinergic REM-active neurons is gated by SubC activity, supporting a mutually excitatory interaction resulting in the generation and maintenance of REM sleep. This mechanism of mutual reinforcement increases the reliability of neural systems to alternate between states of consciousness in a rapid and stable manner (34). In support of these findings, specific optogenetic activation of cholinergic neurons of the laterodorsal (LDT) and pedunculo-pontine tegmentum (PPT) increased the probability of entrance into REM sleep when these neurons were activated during NREM sleep (35).

Another component of the REM-generating circuit is located in the medulla. The dorsal paragigantocellular reticular nucleus (DPGi), a group of GABA-containing neurons, is also REM-active and may inhibit wake-promoting areas; hence, allowing the entrance into REM sleep (36). These medullary neurons are hypothesized to inhibit the LC, dorsal raphe (DR), and part of the ventrolateral periaqueductal gray (vlPAG) (37). Electrical stimulation and pharmacological activation of the DPGi promote REM sleep (38–40).

GABAergic neurons of the vlPAG region are divided into two subpopulations – REM-active and REM-inhibiting. REM-active neurons of this region are thought to silence wake-promoting neurons of the LC and DR. Luppi and colleagues have demonstrated that the vlPAG GABAergic REM-active neurons send projections to these wake-active regions (36, 37, 41). The vlPAG REM-inhibiting neurons send inhibitory inputs to the SubC region and may prevent the activation of the REM-generating circuit (8, 11, 42) (Figures 1 and 2). Drug-induced inhibition and lesions of REM-inhibiting vlPAG neurons lead to lengthening of REM sleep episodes (11, 43). The mutual interaction between brainstem structures (i.e., the SubC, PPT/LDT, vlPAG and DPGi) is responsible for the generation, expression, and maintenance of REM sleep and some of its characteristics.

In addition to the REM-generating network of the brainstem, hypothalamic and forebrain structures project to and influence the core of the REM sleep circuit (44, 45). Melanin-concentrating hormone (MCH) neurons of the lateral hypothalamus (LH) are REM-active, send dense projections to the wake-active serotonergic neurons of the DR, and pharmacological application of MCH in the DR induces a greater number of transitions into REM sleep (46). Optogenetic activation of MCH neurons reduces sleep onset and prolongs the duration of REM sleep (47, 48). MCH neurons also project to wake-promoting histaminergic neurons of the tuberomammilary nucleus (TMN) and noradrenergic cells of the LC, and promote REM sleep through the release of GABA (47–50). Similarly, REM-active neurons of the extended ventrolateral preoptic area (eVLPO) (8) send GABAergic projections to the REM-inhibiting neurons of the vlPAG, thereby freeing the SubC region from its silenced state (51). Finally, REM-active GABAergic neurons of the basal forebrain (BFB) project to the brainstem REM-generating network and may play a role in the regulation of REM sleep (52). Together these observations suggest that REM sleep is controlled by a dispersed network of different transmitter systems; however, we hypothesize that the SubC is the core that coordinates the entrance, maintenance, and exit from REM sleep.

## Cataplexy – Intrusion of REM Sleep Atonia into Wakefulness

Three million people worldwide suffer from narcolepsy (53). A particularly debilitating symptom of this disorder is known as cataplexy, which is the sudden and involuntary reduction or loss of skeletal muscle tone (i.e., motor atonia) during wakefulness. For this reason, cataplexy represents the major impairment of narcoleptic patients and negatively influences their ability to participate in normal day-to-day activities. The severity of cataplexy attacks ranges from transient muscle weakness of the face and/or extremities to complete body paralysis lasting up to several minutes (4). Although cataplexy affects all skeletal muscles aside from the diaphragm and extraocular muscles, its effects are most pronounced on muscles of the neck and face. While the underlying cause of human narcolepsy appears to be either the autoimmune-induced loss of hypocretin neurons or mutation of the hypocretin gene ifself (Figure 1) (54–60), the precise neural mechanisms that trigger cataplexy are unclear.

Cataplexy is thought to result from inappropriate intrusion of REM sleep paralysis into wakefulness (Figure 1) (53, 61, 62). This hypothesis is supported by neuroimaging studies in narcoleptic humans and electrophysiological recordings in narcoleptic dogs, which suggest that the brainstem regions implicated in the control of REM sleep exhibit similar activity during both REM sleep and cataplexy (63, 64). Moreover, the similarity between REM sleep atonia and cataplexy are underscored by the fact that some patients with narcolepsy report hypnagogic hallucinations during cataplectic attacks, which are similar to the vivid dreaming often experienced in REM sleep, and some narcoleptic individuals transition directly into REM sleep from cataplexy (65). Recent but preliminary data shows that activation of the SubC can induce behavioral arrests that resemble cataplexy in orexin knockout mice (66). This observation suggests that cataplexy may result from the pathological recruitment of the SubC circuit that causes REM sleep paralysis. More broadly, this new data suggests that muscle paralysis in REM sleep and cataplexy stem from a common neural mechanism.

Though cataplexy may occur spontaneously, most attacks are precipitated by strong positive emotions such as excited laughter, elation, or surprise (67). Even in healthy individuals, laughter can produce brief muscle weakness, especially in the lower limbs, which is linked to suppression of the Hoffmann reflex – an observation that has given rise to the expression “weak with laughter” (68, 69). Since hypocretin neurons are activated by strong emotions, the loss of this neural population in narcolepsy may destabilize the natural brainstem network that regulates muscle tone and, hence, enable positive emotions to trigger inappropriate motor paralysis in the form of cataplexy (70, 71).

Because the amygdala is intimately involved in processing emotion, it may play a role in the mechanism triggering cataplexy (72). Single-photon emission CT has demonstrated hyperperfusion in the right amygdala during cataplexy (64), and in narcoleptic dogs, amygdala neurons increase activity during cataplectic attacks (63). Furthermore, when the amygdala of hypocretin/orexin knockout mice is lesioned bilaterally, the frequency of cataplectic attacks is significantly reduced (71). Finally, the amygdala is anatomically well-positioned to trigger cataplexy, as it sends extensive GABAergic projections to midbrain and brainstem regions that promote waking muscle tone (i.e., LC, lateral pontine tegmentum (LPT), and the vlPAG) (Figure 1) (71).

The cessation of activity of LC noradrenergic neurons results in the disfacilitation of motor neurons, which in turn contributes to reduce muscle tone (Figure 1) (17). Drugs that increase noradrenaline levels are effective in alleviating cataplexy in humans, dogs and mice (17, 73, 74). Restoration of hypocretin receptors onto DR serotonergic neurons of mice lacking hypocretin receptors decreases the frequency of cataplectic attacks, suggesting that the serotonin signaling system could also be involved in cataplexy (75). However, the firing of DR neurons does not seem to cease during cataplexy, which is in contrast to neurons of the LC (76).

## REM Sleep Behavior Disorder – Breakdown of REM Sleep Circuitry

In contrast to cataplexy, wherein muscle paralysis occurs inappropriately during wakefulness, REM sleep behavior disorder (RBD) is characterized by the absence of normal muscle paralysis during REM sleep (77). Loss of muscle paralysis leaves afflicted individuals able to move while they dream, which frequently results in injury of themselves or their bed partners (78). RBD is thought to arise from damage to the brainstem circuits that mediate REM sleep atonia (Figure 2) (5). Indeed, a phenotype reminiscent of RBD can be generated by physical or genetic lesions of the REM sleep core (i.e., SubC or VMM) in animal models (18, 79). Additionally, brain-imaging studies and postmortem tissue analysis of patients afflicted by RBD have identified lesions encompassing the REM sleep circuitry in the brainstem (80–82).

During normal REM sleep, muscle paralysis is intermittently punctuated by muscle twitches. Since RBD may include an exaggeration of these natural motor events, identifying the functional and neurochemical mechanisms that control this phasic motor activity may help elucidate the pathological process that contributes to the RBD phenotype. Intracellular recording studies show that intermittent release of glutamate excites motoneurons and causes REM sleep muscle twitches (23), through activation of non-NMDA receptors (30). Evidence indicates that the red nucleus (RN), LDT, and PPT nucleus are involved in triggering these twitches (Figure 2) (83–86). Cells located in these nuclei discharge in sync with muscle twitches and other phasic REM sleep phenomena and may be the source of these events. Aside from generating the tonic inhibition of muscle tone during REM sleep, GABA and glycine may help suppress phasic REM sleep activity (18, 20, 21). Indeed, both pharmacological and genetic blockade of GABA and glycine receptors increase muscle twitches during REM sleep (18, 20, 21).

The cholinergic system, which normally functions to promote REM sleep atonia, is altered in RBD patients. Neuroimaging studies reveal that individuals with RBD have significant degradation of cholinergic centers within the brain (24, 87). Taken together, these findings suggest that the amplified motor activity typical of RBD is a consequence of either the over-excitation of the circuit generating twitches or the breakdown of components of the REM sleep muscle atonia circuit (Figure 2).

The excess motor activity that occurs in patients with RBD is often highly coordinated and reflects stereotypical movements seen during wakefulness, an observation that implicates the motor cortex in potentially driving movements associated with RBD. Pyramidal tract neurons control voluntary limb movement and are highly active during both wakefulness and REM sleep (88). However, the destruction of descending corticospinal projection fibers does not abolish REM sleep muscle twitches (89), nor does transection of the brain above the pons in so-called pontine animals or decorticate humans (90). Recently, we have shown that chemogenetic activation of glutamatergic neurons of the RN produces excessive muscle twitching during REM sleep similar to what is observed in RBD (91). Finally, a study in neonatal rats established that muscle twitches during REM sleep are not necessarily the result of cortical activation, but instead drive the activity and development of the motor cortex (92, 93).

A major concern in RBD is that it precedes, in 80% of cases, development of synucleinopathies such as Parkinson’s disease (PD) by several decades (77, 82). This link suggests that neurodegenerative processes initially target the circuits controlling REM sleep and specifically SubC neurons. Subtle motor manifestations, usually bradykinesia, are frequent in idiopathic RBD (94) and quantitative motor tests allow detection of Parkinsonism more than 4 years before the clinical diagnosis of PD (95).

## Future Directions – Characterization of REM Sleep Circuits, and Involvement of the Dopaminergic and Limbic Systems

Although initial studies suggest that SubC neurons generate REM sleep (12), their neurotransmitter and genetic characteristics remain poorly defined. Future studies need to focus on improving characterization of REM-generating circuits and should define how the different neuronal populations of the circuits interact to produce REM sleep. Similarly to how cholinergic cells of the PPT/LDT and glutamatergic cells of the SubC region mutually interact (26, 32, 35), it would be valuable to elucidate the synergistic interaction between other parts of the network (e.g., DPGi, SubC, and MCH). Moreover, we should investigate why such circuits are vulnerable to degeneration in RBD or pathological recruitment in narcolepsy.

The interaction between various neurotransmitter systems regulates REM sleep and its characteristics; however, one system has been understudied in relation to REM sleep and associated disorders – the dopaminergic system. While dopamine levels in the cerebrospinal fluid are highest during wake and lowest in sleep (96), several pieces of evidence indicate a role for the dopamine system in REM sleep control. Most dopamine neurons fire similarly throughout the sleep–wake cycle; however, ventral tegmental area (VTA) neurons fire in burst mode during REM sleep (97). In addition, application of dopamine onto REM-active neurons of the SubC region leads to inhibition of REM sleep or REM sleep without atonia – implying the existence of a REM-inhibiting dopamine cell group (98, 99).

There is strong evidence that dysregulation of the dopamine system contributes to narcolepsy. Dopamine receptor expression is affected in human narcoleptics and is correlated with the severity of cataplexy (100). Drugs used to treat narcolepsy (e.g., modafinil, amphetamine, and clomipramine) affect dopamine system function (101–103). Moreover, drugs that target dopamine receptor activity influence cataplexy in narcoleptic mice (104). Specifically, activation of dopamine D_2_-like receptors increases the frequency of cataplectic attacks in these mice, whereas receptor blockade reduces their occurrence (104). Despite the clear involvement of the dopamine system in mediating cataplexy, the specific part of the dopamine system which contributes to the motor paralysis of cataplexy remains unknown.

Dopamine neurons of the caudal hypothalamus – the A11 cell group – send descending projections to the brainstem and spinal cord; and hence, have been hypothesized to play a role in motor control (98, 99, 105–108). Inhibition of this neuronal region leads to a worsening of cataplectic symptoms in narcoleptic dogs (109). More recent but preliminary work shows that optogentic activation of these dopamine neurons in narcoleptic mice rescues cataplexy within a few seconds of stimulation (110).

The strong link between RBD and PD, a neurodegenerative disorder affecting the dopamine system, suggests that the dopamine system may also be involved in the pathophysiology of RBD. For example, lesions of the dopamine system, using 1-methyl-4-phenyl-1,2,3,6-tet-rahydropyridine hydrochloride (MPTP), have also been shown to produce RBD symptoms in monkeys (111). Immediately after MPTP treatment, monkeys experienced loss of REM sleep motor atonia, despite having normal motor function during wakefulness (i.e., no PD symptoms). Supporting these findings, imaging studies show dopamine cell loss in patients with RBD (112).

Finally, the link between the limbic system and REM sleep circuits has been poorly studied. As mentioned earlier, strong positive emotions trigger cataplexy, which suggests, if cataplexy represents an intrusion of REM sleep into wakefulness, that there is a link between the amygdala (a part of the limbic system) and the REM sleep core. Indeed, anatomical tracing studies have established that the amygdala has both direct and indirect connections with the SubC region (71, 113). Imaging studies demonstrate increased activity in the amygdala during REM sleep (114, 115), and TTX-mediated inhibition of the amygdala decreases both the number and duration of REM sleep episodes (116, 117). Finally, pharmacological studies have shown that neurotransmitters implicated in REM sleep control also affect the amygdala to alter REM sleep expression. For example, GABA_A_ receptor agonism and antagonism of the amygdala produce decreases and increases (respectively) in REM sleep amounts (118), the application of serotonin during NREM sleep produces rapid transitions into REM sleep (119), and cholinergic excitation increases the frequency of REM episodes (120).

## Conclusion

Interaction between the core of the REM-generating circuit and other forebrain, hypothalamic and brainstem structures generate REM sleep and its characteristics (e.g., muscle paralysis). Both direct cholinergic activation (7, 32) and GABAergic inhibition (11, 43) induce the transition into REM sleep by activating SubC glutamatergic neurons. Descending SubC projections activate GABA and glycine release onto motoneurons, producing the paralysis of skeletal muscles in REM sleep (45, 79). Over-expression of REM sleep characteristics or untimely activation of REM sleep circuitry are the pathological causes of several sleep disorders. Abnormal activation of the REM-generating circuit while awake leads to cataplectic attacks in narcoleptic patients (4). Failure to shut down muscles and/or over-expression of motor activity during REM sleep are the primary signs of RBD (5). Finally, further investigation of the genetic and phenotypic characteristics of the REM sleep core system, as well as the interaction between REM sleep circuitry and other parts of the diffuse neural network, which contributes to generating REM sleep, will help shape new specific approaches to treat these REM sleep-associated disorders.

## Conflict of Interest Statement

The authors declare that the research was conducted in the absence of any commercial or financial relationships that could be construed as a potential conflict of interest.
